# Preoperative Cervical Smear Cytology and Clinicopathologic Characteristics in Endometrial Cancer

**DOI:** 10.3390/jcm15176575

**Published:** 2026-08-26

**Authors:** Kubra Cakar Yilmaz, Kevser Arkan, Asli Tugce Sarisoy, Sunullah Soysal

**Affiliations:** 1 Department of Obstetrics and Gynecology, Diyarbakir Gazi Yasargil Training and Research Hospital, Diyarbakir 21070, Turkey; 2 Division of Gynecologic Oncology, Department of Obstetrics and Gynecology, Diyarbakir Gazi Yasargil Training and Research Hospital, Diyarbakir 21070, Turkey; 3 Merz Aesthetics GmbH, 60318 Frankfurt am Main, Germany; 4 Department of Obstetrics and Gynecology, Marmara University Hospital, Istanbul 34899, Turkey

**Keywords:** endometrial cancer, cervical cytology, pap smear, FIGO stage, Müllerian system

## Abstract

**Objectives:** The endometrium and cervix share a common Müllerian origin and may exhibit overlapping biological responses to oncogenic stimuli. However, the clinical significance of preoperative cervical cytology in patients with endometrial cancer remains unclear. This study aimed to investigate the association between preoperative cervical smear cytology and clinicopathologic characteristics, with particular emphasis on advanced FIGO stage (III–IV) as the primary outcome. **Methods:** This retrospective cohort study included 154 patients who underwent primary surgery for histologically confirmed endometrial cancer. Preoperative cervical smear cytology was classified according to the Bethesda System into four categories: normal, low-grade, high-grade, and malignant. For subgroup analyses, low-grade, high-grade, and malignant cytology results were combined and compared with normal cytology. FIGO stage was analyzed both as individual stages (IA–IV) and as early-stage (I–II) versus advanced-stage (III–IV) disease, where appropriate. Continuous variables were summarized as mean ± standard deviation or median (interquartile range), while categorical variables were expressed as *n* (%). Group comparisons were performed using the Pearson chi-square test or Fisher’s exact test, as appropriate, and a two-sided *p* value < 0.05 was considered statistically significant. **Results:** A total of 154 patients with endometrial cancer were included in the analysis. Preoperative cervical smear cytology was normal in 140 patients (90.9%), low-grade in 6 (3.9%), high-grade in 3 (1.9%), and malignant in 5 (3.2%). Comparison across cytology categories demonstrated a significant association between smear cytology and FIGO stage (*p* < 0.001), with high-grade and malignant cytology occurring more frequently in patients with advanced-stage disease. Vaginal cuff involvement was also significantly associated with smear cytology (*p* = 0.001), whereas no significant associations were observed for myometrial invasion, lymphovascular space invasion, cervical stromal involvement, tubo-ovarian involvement, omental involvement, or lymph node metastasis. Subgroup analysis comparing normal and abnormal cytology yielded similar findings, confirming significant associations with FIGO stage (*p* < 0.001) and vaginal cuff involvement (*p* = 0.002). In a multivariable model additionally including cervical cytology, LVSI and deep myometrial invasion were independent predictors of advanced stage (AUC = 0.888), whereas cytology was not statistically significant after adjustment (OR 4.51, *p* = 0.076) and showed poor standalone diagnostic performance (AUC = 0.564, sensitivity 19.4%, specificity 93.5%). All patients with vaginal cuff involvement had advanced-stage disease (Fisher’s exact *p* < 0.001). The cytology–FIGO stage association was not significant in postmenopausal (*p* = 0.065) or endometrioid-only (*p* = 1.00) subgroup analyses. **Conclusions:** Abnormal preoperative cervical smear cytology was significantly associated with advanced FIGO stage and vaginal cuff involvement in patients with endometrial cancer. These findings suggest that cervical cytological abnormalities may reflect overall disease extent rather than individual histopathological risk factors. Given the retrospective design and the limited number of patients with abnormal cytology, these findings should be interpreted cautiously and validated in larger prospective studies. However, cytology was not an independent predictor of advanced stage after multivariable adjustment and performed poorly as a standalone diagnostic marker, suggesting it should not be used alone to assess disease extent.

## 1. Introduction

The synchronous occurrence of primary malignancies within the female genital tract is uncommon, accounting for approximately 0.7% to 1.8% of all gynecologic cancers [1]. The coexistence of multiple primary tumors is thought to reflect shared pathogenic mechanisms rather than a coincidental event. Because tissues derived from a common embryologic origin may respond similarly to carcinogenic, hormonal, genetic, and environmental influences, they may be susceptible to concurrent malignant transformation [2].

In gynecologic oncology, synchronous primary malignancies most commonly involve the endometrium and ovary, whereas the coexistence of primary endometrial and cervical cancers is considerably less frequent [3,4]. To date, the available evidence has been largely limited to isolated case reports and small retrospective series, restricting a comprehensive understanding of the underlying pathogenic mechanisms and their clinical significance [5,6,7].

Endometrial cancer is broadly classified into endometrioid and non-endometrioid (serous, clear cell, mixed, and undifferentiated/dedifferentiated) histologic subtypes, which differ substantially in biological behavior and prognosis; molecular classification is increasingly used alongside histology to guide risk stratification and management. Preoperative assessment of disease extent informs surgical planning and the need for adjuvant therapy. Cervical smear cytology is a well-established, low-cost screening tool for cervical neoplasia and is frequently obtained during preoperative evaluation of patients with endometrial pathology; whether abnormal cytology in this setting provides additional information about the extent of endometrial cancer, beyond its intended purpose of cervical screening, is not well established.

The endometrium and cervix are both derived from the Müllerian ducts, providing a shared embryologic basis that may contribute to their susceptibility to common oncogenic influences. Lauchlan’s theory of the secondary Müllerian system proposes that Müllerian-derived tissues retain a lifelong capacity for metaplastic and neoplastic transformation, particularly following prolonged exposure to carcinogenic stimuli [8]. This concept offers a biologically plausible framework for understanding the development of synchronous malignancies within the female genital tract.

Although HPV is a well-established etiologic factor in cervical carcinogenesis, its role in endometrial cancer is not established. A subset of studies has detected HPV DNA and HPV-associated molecular alterations (e.g., p16 overexpression) in endometrial tumor tissue, particularly in high-grade and non-endometrioid subtypes, but these findings remain preliminary and do not establish causality [9,10,11,12,13,14,15]. This background is presented only as context; HPV status was not evaluated in the present study.

In patients with endometrial cancer, abnormal cervical cytology has been associated with adverse clinicopathologic features, including higher tumor grade, deep myometrial invasion, cervical stromal involvement, and lymphovascular space invasion [16]. These abnormalities may reflect occult synchronous cervical neoplasia, direct tumor extension, or broader epithelial alterations affecting the Müllerian tract. However, the clinical significance of preoperative cervical cytology in endometrial cancer remains uncertain, and its potential value as a marker of disease extent has not been clearly established.

Accordingly, this study was designed to evaluate preoperative cervical smear cytology in patients undergoing surgery for endometrial cancer and to investigate its association with clinicopathologic characteristics. Particular emphasis was placed on its relationship with FIGO stage and other indicators of disease extent to determine whether preoperative cervical cytology may provide clinically relevant information beyond routine cervical cancer screening.

## 2. Materials and Methods

This retrospective cohort study was conducted at the Department of Gynecologic Oncology, Marmara University Faculty of Medicine. Women aged 35–70 years who underwent primary surgical treatment for histologically confirmed endometrial cancer between February 2018 and July 2020 were identified through the institutional surgical and pathology databases.

All patients underwent cervical cytology within a 3-month interval before surgery. Patients were excluded if preoperative cervical smear cytology was unavailable, if they had a previous diagnosis of cervical cancer, synchronous gynecologic malignancies other than endometrial cancer, had received neoadjuvant therapy, or had incomplete clinicopathologic data that precluded analysis. After applying the eligibility criteria, 154 patients were included in the final study cohort.

The study protocol was approved by the Marmara University Clinical Research Ethics Committee. The requirement for informed consent was waived because of the retrospective study design. All procedures were performed in accordance with the ethical principles of the Declaration of Helsinki.

### 2.1. Exposure and Outcome Definitions

The primary exposure variable was preoperative cervical smear cytology, which was classified according to the Bethesda System for Reporting Cervical Cytology. Cytologic findings were categorized as negative for intraepithelial lesion or malignancy (NILM), low-grade abnormalities (ASC-US or LSIL), high-grade abnormalities (ASC-H, HSIL, or AGC), or malignant cytology suggestive of carcinoma or adenocarcinoma. Classification was based exclusively on cytologic findings; histopathologic diagnoses, including cervical intraepithelial neoplasia, were not considered to avoid misclassification.

For subgroup analyses, cytology results were further dichotomized into normal (NILM) and abnormal (low-grade, high-grade, or malignant cytology). The primary outcome was the association between cervical cytology and FIGO stage, evaluated both across the four cytologic categories and after dichotomization into normal and abnormal cytology. Advanced disease was defined as FIGO stage III–IV.

Secondary outcomes included the associations between cervical cytology and other clinicopathologic characteristics, including the depth of myometrial invasion, lymphovascular space invasion (LVSI), cervical stromal involvement, vaginal cuff involvement, tubo-ovarian involvement, omental involvement, lymph node metastasis, and demographic characteristics.

### 2.2. Surgical and Pathological Assessment

All patients underwent primary surgical treatment consisting of total hysterectomy with or without bilateral salpingo-oophorectomy, with surgical staging performed according to institutional practice and contemporary international guidelines.

All cytologic and histopathologic reports were prepared by a single dedicated gynecologic pathologist/cytopathologist over the study period, and an additional, dedicated pathology re-review was performed specifically for this study. All cases were retrospectively reclassified according to the 2023 FIGO criteria for this study.

Histopathologic findings were obtained from the final pathology reports. Histologic subtype and tumor grade were classified according to the World Health Organization (WHO) Classification of Female Genital Tumours, and disease stage was assigned using the 2023 International Federation of Gynecology and Obstetrics (FIGO) staging system.

Demographic and clinicopathologic data were retrospectively extracted from electronic medical records and final pathology reports. Variables collected included age at diagnosis, parity, menopausal status, preoperative cervical smear cytology, histologic subtype, FIGO stage, depth of myometrial invasion, lymphovascular space invasion (LVSI), cervical stromal involvement, vaginal cuff involvement, tubo-ovarian involvement, omental involvement, lymph node metastasis, and distant metastasis.

### 2.3. Statistical Analysis

Statistical analyses were performed using Python (version 3.12; Python Software Foundation, Wilmington, DE, USA). Continuous variables are presented as mean ± standard deviation or median (interquartile range), as appropriate according to data distribution, whereas categorical variables are presented as frequencies and percentages. Comparisons of continuous variables were performed using one-way analysis of variance (ANOVA) or the Kruskal–Wallis test, as appropriate. Categorical variables were compared using the Pearson chi-square test or Fisher’s exact test, as appropriate. All statistical tests were two-sided, and a *p* value < 0.05 was considered statistically significant.

A multivariable logistic regression model was constructed to identify independent predictors of advanced-stage disease (FIGO stage III–IV), incorporating age, histologic risk category (low-risk: endometrioid carcinoma grade 1–2; high-risk: grade 3 endometrioid, serous, clear cell, mixed, or undifferentiated/dedifferentiated carcinoma), lymphovascular space invasion (LVSI), depth of myometrial invasion (≥50% vs. <50%/none), and cervical cytology (abnormal vs. normal). Odds ratios (ORs) with 95% confidence intervals (CIs) were calculated. Model discrimination was assessed using the area under the receiver operating characteristic curve (AUC). The diagnostic performance of cervical cytology alone for advanced-stage disease was additionally evaluated using sensitivity, specificity, positive predictive value (PPV), negative predictive value (NPV), and AUC. Subgroup analyses examining the association between cytology (normal vs. abnormal) and FIGO stage group (early vs. advanced) were repeated among postmenopausal patients and among patients with endometrioid carcinoma, using Fisher’s exact test. Patients with missing data for any variable in a given analysis were excluded from that analysis (complete-case analysis). Consequently, the sample size varied across analyses according to the availability of complete data for the variables included in each model.

Given the limited number of patients in the low-grade, high-grade, and malignant cytology groups, the results should be interpreted with appropriate caution.

## 3. Results

A total of 154 patients who underwent primary surgical treatment for histologically confirmed endometrial cancer were included in the study. The mean age was 59.8 ± 9.7 years (median, 60 years; interquartile range [IQR], 53.0–66.0). Most patients were postmenopausal (87.7%), and endometrioid carcinoma was the predominant histologic subtype (85.7%). The baseline demographic and clinicopathologic characteristics of the study population are summarized in Table 1.

Preoperative cervical smear cytology was normal in 140 patients (90.9%), low-grade in 6 (3.9%), high-grade in 3 (1.9%), and malignant in 5 (3.2%).

Comparisons across the four cytology categories are presented in Table 2. Cervical smear cytology was significantly associated with FIGO stage (*p* < 0.001), with high-grade and malignant cytology occurring more frequently in patients with advanced-stage disease. Vaginal cuff involvement also differed significantly among the cytology groups (*p* = 0.001). In contrast, no significant associations were observed between cytology category and myometrial invasion, lymphovascular space invasion (LVSI), cervical stromal involvement, tubo-ovarian involvement, omental involvement, or lymph node metastasis.

The subgroup analysis comparing normal and abnormal cytology is summarized in Table 3. Abnormal cytology was significantly associated with advanced FIGO stage (*p* < 0.001) and vaginal cuff involvement (*p* = 0.002). No significant differences were identified with respect to myometrial invasion, LVSI, cervical stromal involvement, tubo-ovarian involvement, or omental involvement.

Vaginal cuff involvement was cross-tabulated against FIGO stage group (early stage I–II vs. advanced stage III–IV; Table 4) to clarify the relationship between disease extent and vaginal cuff status across the entire cohort (*N* = 154). Among the 7 patients with vaginal cuff involvement identified on final pathology, 3 had stage III disease and 4 had stage IV disease (0 patients in stage IA, IB, or II). Consequently, all patients with vaginal cuff involvement had advanced-stage (FIGO III–IV) disease, whereas none of the early-stage (FIGO I–II) patients had vaginal cuff involvement (Fisher’s exact *p* < 0.001).

In the multivariable logistic regression model (*n* = 123 patients with complete data for all included covariates; 28 with advanced-stage disease), LVSI (OR 20.00, 95% CI 5.46–73.31, *p* < 0.001) and deep myometrial invasion ≥50% (OR 6.88, 95% CI 1.92–24.66, *p* = 0.003) were independently associated with advanced FIGO stage. Abnormal cervical cytology showed a positive but not statistically significant independent association (OR 4.51, 95% CI 0.85–23.87, *p* = 0.076) after adjustment for these established factors; age (OR 0.96, 95% CI 0.90–1.02, *p* = 0.184) and high-risk histologic subtype (OR 1.96, 95% CI 0.60–6.42, *p* = 0.268) were not statistically significant predictors (Table 5). The model showed good overall discrimination for advanced-stage disease (AUC = 0.888).

The standalone diagnostic performance of cervical cytology (abnormal vs. normal) for advanced-stage disease was modest (*n* = 138; Table 6). Cytology showed high specificity (93.5%) and a reasonably high negative predictive value (80.0%) but low sensitivity (19.4%) and a moderate positive predictive value (46.2%); overall discrimination was poor (AUC = 0.564, close to chance). Together with its non-significant independent contribution in the multivariable model above, these findings indicate that, while abnormal cytology is statistically associated with advanced-stage disease in unadjusted analyses (Table 2 and Table 3), it performs poorly as a standalone diagnostic marker and does not clearly add independent information once established prognostic factors (LVSI, depth of myometrial invasion) are taken into account.

In the subgroup analyses, the association between cervical cytology and FIGO stage group did not reach statistical significance when restricted to postmenopausal patients (*n* = 117: 5/11 advanced-stage patients with abnormal cytology vs. 21/106 early-stage patients with abnormal cytology; Fisher’s exact *p* = 0.065) or when restricted to patients with endometrioid carcinoma (*n* = 95: 1/14 advanced-stage patients with abnormal cytology vs. 5/81 early-stage patients with abnormal cytology; Fisher’s exact *p* = 1.00).

Regarding histologic subtype, endometrioid carcinoma accounted for the large majority of cases (132/154, 85.7%), while non-endometrioid histologies were less frequent (serous carcinoma, 8/154, 5.2%; clear cell carcinoma, 2/154, 1.3%; mixed carcinoma, 3/154, 1.9%; undifferentiated/other, 9/154, 5.8%; Table 1). Given this distribution, the cohort was not powered to detect differences in the cytology–FIGO stage association across individual non-endometrioid subtypes, and the predominance of endometrioid histology should be considered when interpreting the overall findings and comparing them with cohorts that include a larger proportion of non-endometrioid tumors, such as that of Roelofsen et al. [17].

## 4. Discussion

In this retrospective study, analyses across the four cervical smear cytology categories and the subgroup comparison of normal versus abnormal cytology consistently demonstrated significant associations with advanced FIGO stage and vaginal cuff involvement. These findings suggest that abnormal cervical cytology may reflect overall disease extent rather than individual histopathologic risk factors, supporting its potential value as an adjunctive marker in preoperative risk assessment.

Although HPV is a well-established etiologic factor in cervical carcinogenesis, its role in endometrial cancer remains uncertain. Detection of HPV DNA in a subset of endometrial carcinomas has led to hypotheses involving field cancerization, ascending infection, or shared oncogenic mechanisms, although these remain speculative [8]. Because HPV testing was not performed in the present study, no conclusions regarding its biological role can be drawn. Instead, the observed association between abnormal cervical cytology and advanced disease may reflect the shared embryologic origin of the cervix and endometrium or the presence of occult synchronous cervical pathology.

Our findings are broadly consistent with those reported by Roelofsen et al. [17], who observed that abnormal cervical cytology was more common in patients with less favorable clinicopathologic characteristics, particularly non-endometrioid tumors. Similarly, we found a significant association between abnormal cytology and advanced FIGO stage. However, unlike their study, abnormal cytology was not associated with myometrial invasion or LVSI in our cohort. This difference may be related to the predominance of endometrioid carcinoma in our study population, underscoring the influence of histologic subtype on the relationship between cervical cytology and disease characteristics.

From a clinical perspective, abnormal preoperative cervical smear cytology in patients with endometrial cancer should prompt careful evaluation for synchronous cervical pathology and may alert clinicians to the possibility of more advanced disease. Although cervical cytology should not be used in isolation to guide management, it may provide complementary information for preoperative risk assessment.

Our findings differ somewhat from those reported by Brown et al. [18] and DuBeshter et al. [19], both of whom described associations between abnormal cervical cytology and unfavorable clinicopathologic characteristics, including deep myometrial invasion, higher tumor grade, advanced-stage disease, and poorer clinical outcomes. In contrast, abnormal cytology in our cohort was significantly associated with advanced FIGO stage and vaginal cuff involvement but not with myometrial invasion or lymphovascular space invasion. These differences may reflect variations in patient selection, histologic subtype distribution, cohort characteristics, or study design. Although cervical cytology should not be used in isolation to guide clinical decision-making, it may provide complementary information during preoperative risk assessment. Future prospective studies integrating cervical cytology with HPV genotyping and molecular profiling are warranted to further clarify the biological basis and clinical significance of these associations [4].

To our knowledge, few studies have examined the relationship between preoperative cervical cytology and specific patterns of disease spread, including vaginal cuff involvement, in surgically treated endometrial cancer. Our findings suggest that abnormal cervical cytology is associated with advanced disease rather than isolated histopathologic features, supporting its potential role as a readily available adjunct in preoperative risk assessment. Future prospective studies incorporating molecular profiling and HPV genotyping are needed to further clarify the biological basis of these associations.

Our findings are also consistent with contemporary concepts of endometrial cancer risk stratification. The 2023 FIGO staging system emphasizes accurate assessment of disease extent to improve prognostic classification and guide individualized management [20], whereas the 2020 WHO Classification highlights the biological heterogeneity of endometrial cancer across histologic subtypes [21]. In this context, the association between abnormal cervical cytology and advanced FIGO stage, despite the absence of significant associations with individual histopathologic risk factors, suggests that cervical cytology may reflect overall tumor burden rather than specific microscopic features.

We additionally examined independent predictors of advanced-stage disease using multivariable logistic regression incorporating cervical cytology alongside established prognostic factors. LVSI and deep myometrial invasion remained independent predictors of advanced FIGO stage, consistent with their established prognostic role in endometrial cancer. Abnormal cervical cytology showed a positive but statistically non-significant independent association with advanced stage (OR 4.51, *p* = 0.076) after adjustment, and its standalone diagnostic performance was poor (AUC = 0.564), driven mainly by low sensitivity (19.4%) despite high specificity (93.5%). Taken together, these findings suggest that, although abnormal cytology is associated with advanced-stage disease in unadjusted comparisons, it does not perform well as an independent or standalone diagnostic marker once established pathologic risk factors are accounted for; a positive association may in part reflect confounding by, or correlation with, LVSI and deep myometrial invasion rather than an independent biological relationship. Given the wide confidence interval around the cytology OR, we cannot rule out a true independent effect that this sample size was underpowered to detect; a larger, prospectively designed study would be needed to resolve this. On this basis, we do not propose preoperative cervical cytology as a standalone tool for altering surgical planning or adjuvant treatment decisions; given its high specificity and NPV, an abnormal result may still reasonably prompt closer preoperative evaluation for more extensive disease, to be interpreted alongside imaging and other established preoperative assessments, but a normal result should not be taken as reassurance against advanced disease given its low sensitivity. The cross-tabulation of vaginal cuff involvement by FIGO stage (Table 4) showed that all 7 patients with cuff involvement had advanced-stage (FIGO III–IV) disease, supporting the interpretation that vaginal cuff involvement in this cohort reflects overall disease extent rather than an isolated, independent finding.

In the subgroup analyses, the association between cervical cytology and FIGO stage did not reach statistical significance among postmenopausal patients (*p* = 0.065) or among patients with endometrioid carcinoma (*p* = 1.00). The postmenopausal result approached, but did not reach, significance and, given the modest subgroup size, should be interpreted as a trend rather than a confirmed association. The absence of a significant association within the endometrioid subgroup is consistent with our overall interpretation, discussed above, that the predominance of endometrioid histology in this cohort may partly explain why our findings differ from those of Roelofsen et al. [17], who reported stronger associations in a cohort with a larger proportion of non-endometrioid tumors.

This study has several limitations. First, its retrospective single-center design precludes causal inference and may have introduced selection and information bias. Second, the small number of patients with low-grade, high-grade, and malignant cervical cytology (14/154, 9.1%) limited the statistical power of subgroup and multivariable analyses involving cytology; the wide confidence interval around the adjusted odds ratio for cytology (OR 4.51, 95% CI 0.85–23.87) reflects this limited power, and a true independent association cannot be excluded. Nevertheless, the study also has important strengths, including surgically confirmed staging, comprehensive pathologic evaluation, and a uniformly managed cohort from a tertiary gynecologic oncology center. Therefore, the findings should be interpreted as hypothesis-generating and require validation in larger prospective multicenter studies.

## 5. Conclusions

Abnormal preoperative cervical smear cytology was significantly associated with advanced FIGO stage and vaginal cuff involvement in unadjusted analyses, and vaginal cuff involvement was observed exclusively among patients with advanced-stage disease. However, in a multivariable model that also included cervical cytology, LVSI and deep myometrial invasion were the independent predictors of advanced stage (cytology OR 4.51, *p* = 0.076), and cytology performed poorly as a standalone diagnostic marker (AUC = 0.564, sensitivity 19.4%). The association between cytology and advanced stage did not reach significance in subgroup analyses restricted to postmenopausal patients (*p* = 0.065) or patients with endometrioid carcinoma (*p* = 1.00). Taken together, these findings suggest that, while abnormal cervical cytology is associated with advanced-stage disease at the univariate level, it should not be used as a standalone or independent marker of disease extent in endometrial cancer; its main potential value may lie in prompting closer evaluation for more extensive disease, given its high specificity, rather than as a diagnostic test in its own right. Given the retrospective design and the limited number of patients with abnormal cytology, these findings should be confirmed in larger prospective studies.

## Figures and Tables

**Table 1:** Baseline demographic and clinicopathologic characteristics of the study population (N=154).

Parameter	Summary Statistics
Continuous Variables	Mean ± SD/Median (IQR)
- Age (years)	59.8 ± 9.7/60.0 (53.0–66.0)
- Parity	2.1 ± 1.3/2.0 (1.0–3.0)
Categorical Variables	n (%)
Smear Cytology	
- Normal	140 (90.9%)
- Low-grade (ASC-US/LSIL)	6 (3.9%)
- High-grade (HSIL/AGC)	3 (1.9%)
- Malignant	5 (3.2%)
Menopausal Status	
- Premenopausal	19 (12.3%)
- Postmenopausal	135 (87.7%)
Histologic Type	
- Endometrioid carcinoma	132 (85.7%)
- Serous carcinoma	8 (5.2%)
- Clear cell carcinoma	2 (1.3%)
- Mixed carcinoma	3 (1.9%)
- Undifferentiated/others	9 (5.8%)
FIGO Stage (2009/2023)	
- Stage IA	102 (66.2%)
- Stage IB	31 (20.1%)
- Stage II	9 (5.8%)
- Stage III	8 (5.2%)
- Stage IV	4 (2.6%)
Myometrial Invasion	
- <50% (None/Superficial)	103 (66.9%)
- ≥50% (Deep)	51 (33.1%)
Lymphovascular Space Invasion (LVSI)	
- Absent	101 (65.6%)
- Present	53 (34.4%)
Cervical Stromal Involvement	
- Absent	137 (89.0%)
- Present	17 (11.0%)
Vaginal Cuff Involvement	
- Absent	147 (95.5%)
- Present	7 (4.5%)
Tubo-ovarian Involvement	
- Absent	142 (92.2%)
- Present	12 (7.8%)
Omental Involvement	
- Absent	148 (96.1%)
- Present	6 (3.9%)
Lymph Node Metastasis	
- Absent	143 (92.9%)
- Present	11 (7.1%)
Distant Metastatic Disease	
- Absent	150 (97.4%)
- Present	4 (2.6%)

Note: SD, standard deviation; IQR, interquartile range. Categorical data are presented as numbers and percentages (n,%). Percentages may not sum to 100% due to rounding.

**Table 2:** Comparison of clinicopathologic characteristics according to preoperative cervical smear cytology (*N* = 154).

Parameter	Normal (*n* = 140)	Low-Grade (*n* = 6)	High-Grade (*n* = 3)	Malignant (*n* = 5)	*p* Value
FIGO stage, *n* (%)					<0.001
Stage IA	100 (71.4)	2 (33.3)	0 (0.0)	0 (0.0)	
Stage IB	28 (20.0)	2 (33.3)	1 (33.3)	0 (0.0)	
Stage II	7 (5.0)	1 (16.7)	1 (33.3)	0 (0.0)	
Stage III	4 (2.9)	1 (16.7)	1 (33.3)	2 (40.0)	
Stage IV	1 (0.7)	0 (0.0)	0 (0.0)	3 (60.0)	
Vaginal cuff involvement, *n* (%)					0.001
Absent	137 (97.9)	5 (83.3)	2 (66.7)	3 (60.0)	
Present	3 (2.1)	1 (16.7)	1 (33.3)	2 (40.0)	
Myometrial invasion, *n* (%)					0.113
<50% myometrial invasion	96 (68.6)	4 (66.7)	2 (66.7)	1 (20.0)	
≥50% myometrial invasion	44 (31.4)	2 (33.3)	1 (33.3)	4 (80.0)	
Cervical stromal involvement, *n* (%)					0.379
Absent	126 (90.0)	5 (83.3)	2 (66.7)	4 (80.0)	
Present	14 (10.0)	1 (16.7)	1 (33.3)	1 (20.0)	
Lymphovascular space invasion (LVSI), *n* (%)					0.734
Absent	92 (65.7)	4 (66.7)	2 (66.7)	3 (60.0)	
Present	48 (34.3)	2 (33.3)	1 (33.3)	2 (40.0)	
Tubo-ovarian involvement, *n* (%)					0.402
Absent	131 (93.6)	5 (83.3)	2 (66.7)	4 (80.0)	
Present	9 (6.4)	1 (16.7)	1 (33.3)	1 (20.0)	
Omental involvement, *n* (%)					0.558
Absent	136 (97.1)	6 (100.0)	2 (66.7)	4 (80.0)	
Present	4 (2.9)	0 (0.0)	1 (33.3)	1 (20.0)	
Lymph node metastasis, *n* (%)					0.289
Absent	132 (94.3)	5 (83.3)	2 (66.7)	4 (80.0)	
Present	8 (5.7)	1 (16.7)	1 (33.3)	1 (20.0)	

FIGO, International Federation of Gynecology and Obstetrics; LVSI, lymphovascular space invasion. Categorical variables were compared using the Pearson chi-square test or Fisher’s exact test, as appropriate.

**Table 3:** Comparison of clinicopathologic characteristics between patients with normal and abnormal cervical smear cytology (*N* = 154).

Parameter	Normal Cytology (*n* = 140)	Abnormal Cytology * (*n* = 14)	*p* Value
FIGO stage, *n* (%)			<0.001
Stage I–II	135 (96.4)	7 (50.0)	
Stage III–IV	5 (3.6)	7 (50.0)	
Myometrial invasion, *n* (%)			0.224
<50% myometrial invasion	96 (68.6)	7 (50.0)	
≥50% myometrial invasion	44 (31.4)	7 (50.0)	
Lymphovascular space invasion (LVSI), *n* (%)			0.881
Absent	92 (65.7)	9 (64.3)	
Present	48 (34.3)	5 (35.7)	
Cervical stromal involvement, *n* (%)			0.471
Absent	126 (90.0)	11 (78.6)	
Present	14 (10.0)	3 (21.4)	
Vaginal cuff involvement, *n* (%)			0.002
Absent	137 (97.9)	10 (71.4)	
Present	3 (2.1)	4 (28.6)	

* Abnormal cytology includes low-grade, high-grade, and malignant cervical smear results. FIGO, International Federation of Gynecology and Obstetrics; LVSI, lymphovascular space invasion. Values are presented as *n* (%). Categorical variables were compared using Pearson’s chi-square test or the Fisher–Freeman–Halton exact test, as appropriate.

**Table 4:** Vaginal cuff involvement according to FIGO stage group (*N* = 154).

Vaginal Cuff İnvolvement	Early Stage (FIGO I–II), *n* (%)	Advanced Stage (FIGO III–IV), *n* (%)	*p* Value
Absent (*n* = 147)	142 (96.6%)	5 (3.4%)	<0.001
Present (*n* = 7)	0 (0.0%)	7 (100.0%)	

Data are presented as *n* (%). Categorical variables were compared using Fisher’s exact test. Among the 7 patients with vaginal cuff involvement, 3 had FIGO stage III and 4 had FIGO stage IV disease.

**Table 5:** Multivariable logistic regression for advanced FIGO stage (III–IV).

Variable	OR	95% CI	*p* Value
Age (years)	0.96	0.90–1.02	0.184
High-risk histology *	1.96	0.60–6.42	0.268
LVSI (present vs. absent)	20.00	5.46–73.31	<0.001
Myometrial invasion ≥ 50%	6.88	1.92–24.66	0.003
Abnormal cervical cytology	4.51	0.85–23.87	0.076

* High-risk histology: grade 3 endometrioid, serous, clear cell, mixed, or undifferentiated/dedifferentiated carcinoma; low-risk: grade 1–2 endometrioid carcinoma. OR, odds ratio; CI, confidence interval; LVSI, lymphovascular space invasion. Model AUC = 0.888, *n* = 123 (28 with advanced-stage disease).

**Table 6:** Diagnostic performance of abnormal cervical cytology for advanced FIGO stage (III–IV).

AUC	Sensitivity	Specificity	PPV	NPV
0.564	19.4%	93.5%	46.2%	80.0%

PPV, positive predictive value; NPV, negative predictive value. *n* = 138 patients with complete cytology and FIGO stage data.

## Data Availability

The datasets used and/or analyzed during the current study are available from the corresponding author upon reasonable request.

## Abbreviations

AGC	Atypical glandular cell
ASC-US	Atypical squamous cell of undetermined significance
CIN	Cervical intraepithelial neoplasia
FIGO	International Federation of Gynecology and Obstetrics
HPV	Human papillomavirus
HSIL	High-grade squamous intraepithelial lesion
IQR	Interquartile range
LSIL	Low-grade squamous intraepithelial lesion
LVSI	Lymphovascular space invasion
Pap	Papanicolaou
WHO	World Health Organization
